# Can medical students identify a potentially serious acetaminophen dosing error in a simulated encounter? a case control study

**DOI:** 10.1186/s12909-015-0288-3

**Published:** 2015-02-11

**Authors:** Robert A Dudas, Michael A Barone

**Affiliations:** Department of pediatrics, Johns Hopkins University School of Medicine, Baltimore, MD USA

## Abstract

**Background:**

In an effort to assess medical students’ abilities to identify a medication administration error in an outpatient setting, we designed and implemented a standardized patient simulation exercise which included a medication overdose.

**Methods:**

Fourth year medical students completed a standardized patient (SP) simulation of a parent bringing a toddler to an outpatient setting. In this case-control study, the majority of students had completed a patient safety curriculum about pediatric medication errors prior to their SP encounter. If asked about medications, the SP portraying a parent was trained to disclose that she was administering acetaminophen and to produce a package with dosing instructions on the label. The administered dose represented an overdose. Upon completion, students were asked to complete an encounter note.

**Results:**

Three hundred forty students completed this simulation. Two hundred ninety-one students previously completed a formal patient safety curriculum while 49 had not. A total of two hundred thirty-four students (69%) ascertained that the parent had been administering acetaminophen to their child. Thirty-seven students (11%) determined that the dosage exceeded recommended dosages. There was no significant difference in the error detection rates of students who completed the patient safety curriculum and those who had not.

**Conclusions:**

Despite a formal patient safety curriculum concerning medication errors, 89% of medical students did not identify an overdose of a commonly used over the counter medication during a standardized patient simulation. Further educational interventions are needed for students to detect medication errors. Additionally, 31% of students did not ask about the administration of over the counter medications suggesting that students may not view such medications as equally important to prescription medications. Simulation may serve as a useful tool to assess students’ competency in identifying medication administration errors.

## Background

According to data from the American Association of Poison Control Centers, 11% of children younger than 6 years exposed to pharmaceuticals experience a medication error [1]. Recent findings indicate that most preventable adverse drug events in pediatric outpatients are attributable to errors in medication administration [2]. Errors occur frequently; at least 50% of parents make errors when dosing liquid medications [3,4]. Although acetaminophen is available as a nonprescription over-the-counter (OTC) medication and is generally considered safe for children, acetaminophen is one of the most frequently implicated pharmaceuticals involved in cases reported to the American Association of Poison Control Center’s National Poison Data System [5]. Researchers have demonstrated that acetaminophen-dosing errors occur more than 25% of the time and that this percentage increases to more than 50% of the time if patients are Spanish-speaking, despite caregivers receiving written dosing instructions [6]. This is particularly troubling as acetaminophen has a narrow therapeutic index with overdosage linked to hepatotoxicity and death [6,7]. Recently, a US Food and Drug Administration advisory board was convened to focus on strategies to decrease acetaminophen-related toxic exposure [8] and manufacturers have voluntarily moved towards a single concentration of pediatric liquid acetaminophen [9].

Pediatric medication errors are an important patient safety concern. Most of the research to date has been skewed towards prescribing errors despite growing evidence that the process of dispensing and administering medication are just as error-prone, and possibly more so, than prescribing [10]. In contrast to adults, children rely much more on manual compounding of liquid medications and administration by caregivers. Additionally, much of the evidence for identifying and mitigating medication errors is obtained from inpatient settings where less attention is paid to medications that are available over-the-counter and administered by caregivers. Errors associated with medication administration by providers, as well as caregivers, represent an important opportunity for preventative healthcare as they are avertible events.

As a response to the growing appreciation and understanding of medical error, medical schools are developing and evaluating patient safety curricula to prepare future physicians for practice. We have previously described our patient safety curriculum in which students were asked to identify and investigate a medication error during their clinical experiences in pediatrics [11]. Our prior investigation demonstrated that students were able to independently identify actual medication errors during their clinical clerkship in pediatrics and this was associated with positive changes in attitudes toward patient safety. In an effort to assess their ability to identify medication errors when they were not explicitly instructed to search for them, we designed a standardized patient (SP) exercise with an embedded medication administration error. Other studies of medical students have found that they were poor at identifying prescribing errors in a simulated setting [12]. Information about medical students’ abilities to identify medication errors may provide data for the continued development of patient safety curricula. The objective structured clinical examination (OSCE) is a useful method for the evaluation of patient safety competencies and has been previously used to examine the patient safety competencies of undergraduate medical trainees [13]. The objective of this study was to compare medical students’ identification of a medication administration error embedded into a simulation-based OSCE. We specifically compared students who had previously completed a patient safety curriculum focused on medication administration errors with those that had not.

## Methods

### Learner population

The Johns Hopkins University School of Medicine (JHUSOM) is located in Baltimore, Maryland. Each year approximately 120 medical students matriculate into a 4-year-long program, and all students are required to complete and pass a multi-station standardized patient exercise termed the Comprehensive Clinical Skills Examination (CCSE) of which 2 stations contain pediatric content. The CCSE is administered between the 3^rd^ and 4^th^ years of training and lasts approximately 7 hours. All students also complete a patient safety curriculum during their Pediatric Clerkship [11]. A small group of students complete the CCSE examination without having yet completed the Pediatric Clerkship and as a result, they complete the CCSE prior to having completed the patient safety curriculum providing the opportunity to perform this case-control study.

We collected and evaluated student performance data for 3 consecutive academic years (2010-2011, 2011-2012 and 2012-2013). This study was based at the JHUSOM Simulation Center. The Johns Hopkins Institutional Review Board deemed this curriculum improvement study exempt.

### Comprehensive clinical skills examination

The CCSE requires students to rotate through 10 stations (plus 1 pilot case) involving an SP encounter, and are expected to take a patient history, perform a focused physical examination (in 8 cases), and document a differential diagnosis and management plan. The entire exam takes approximately seven hours to complete. Prior to taking this comprehensive examination, students have had a number (n = 10-20) of both formative and summative standardized patient exercises as part of the clinical curriculum, although none are as long as the CCSE.

During the examination, student encounters are displayed on a video screen at a central proctoring station. Two cameras record the encounter and these recordings are archived digitally using B-line Medical software (Washington, DC). Each student case is allocated 25 minutes of time – divided as 15 minutes for the patient encounter and 10 minutes for recording a structured note, which the student types into a B-Line Medical template. The structured note includes specific headings for History, Physical exam, Data Interpretation with Supporting Evidence, and Management Plan. The notes are scored using a 10-point grading rubric. Aside from the documentation note, SPs assess students in three domains of the examination – History, Physical Exam and Interpersonal Communication Skills according to completion of items on a predefined checklist (scored “done correctly” = 1, or “not done” = 0), which SPs submit electronically immediately after each encounter. Students must pass all four domains of the examination to pass the exam. Minimum percentage passing scores for each of the four domains were set by a standard setting panel using the Hofstee method. The CCSE is a high-stakes exam because a passing score is required for graduation. The failure rate on the first attempt for the examination ranges between two and five percent of students. Content is based on domains and specifications defined by the National Board of Medical Examiners and used for the United States Medical Licensing Examination Step 2 Clinical Skills exam. All cases and checklists undergo extensive pilot testing and psychometric analysis before contributing to a student’s overall score. For the 2010 academic year overall reliability for the CCSE (Chronbach’s alpha: 0.68) was similar to other high stakes examinations such as those from the National Board of Medical Examiners [14]. The CCSE uses video monitors in addition to the SP in the examination room and after three encounters the video monitor goes over the checklist with the room SP and any items that are not agreed upon result in the student being awarded credit for that item.

### Simulation-based objective structured clinical examination

This OSCE station presents a parent (SP) concerned about her toddler’s respiratory symptoms and includes a pediatric medication administration error in the history. For this particular station, students were to obtain a medical history from the SP, perform an ear examination on a partial task trainer, view a video of a tympanic membrane obtained via video otoscopy and record a structured note. The specific students’ tasks for the OSCE are listed in Table 1. Each year two SPs were trained to portray the role of parent for this OSCE. At the completion of each encounter the SP completed a checklist. An additional independent monitor also observed the encounter by video and completed the same checklist. Thus, we were able to assess the inter-rater reliability and report an intraclass correlation coefficient (ICC) of 0.86. An ICC of >0.75 indicates good agreement among raters’ scores and thus good reliability.

**Table 1:** **Student tasks for the observed structured clinical examination**

In exam room	Outside exam room
Obtain the relevant history (including history of present illness, past medical history, medications and social history) from the parent (SP) of a toddler with respiratory symptoms.	Document the history, document and interpret the video otoscopic findings, document an appropriate assessment and plan.
Correctly perform an otoscopic examination of a child’s tympanic membrane (via the use of a partial task trainer).	
View a video on a 32 inch display monitor demonstrating insufflation of a tympanic membrane obtained via a video-otoscope.	

### Medication error

**Figure 1:**
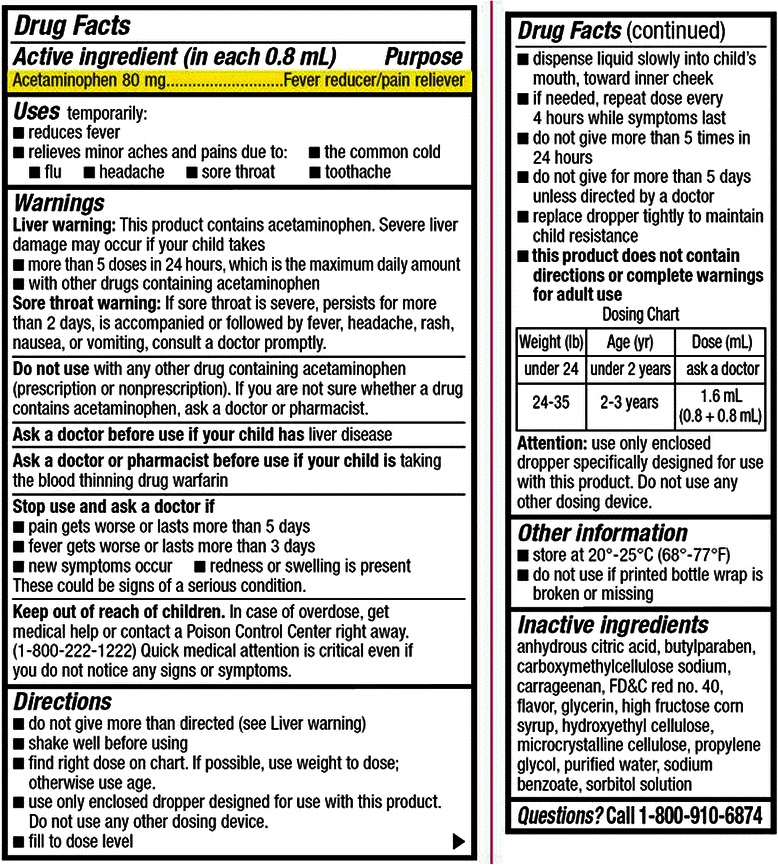
**Packaging as presented to students during simulation.**

For this OSCE a female SP portrayed the parent of a 2-year-old toddler, weighing12 kg, presenting for an acute care visit to a primary care clinic. The chief complaint reported by the SP was, “runny nose, cough and congestion”. If, and when, prompted the SP reported that she had been giving the toddler “2 teaspoonfuls” of infant acetaminophen (80 mg/0.8 ml) several times a day for the past 5 days. The SP would then produce a bottle of infant acetaminophen from her purse and place it on the counter for the remainder of the encounter (Figure 1) providing the opportunity for the student to review recommended dosing instructions on the label should he/she choose to.

This 1000 mg dose of acetaminophen yields a dose of 83 mg/kg representing approximately a 6-to-8 fold overdose. As there was no actual child in the room, the partial task trainer served as the only basis for testing the students’ psychomotor skills of otoscopy. Upon completion of the history, students were directed to look at a video monitor revealing a video clip of a dull tympanic membrane with air fluid levels which was being insufflated. Students were asked to describe the tympanic membrane and relate it to the child’s diagnosis.

### Outcome measures

Immediately after the encounter, SPs were asked to complete a 13-item checklist which included three questions regarding acetaminophen administration. The first item reported whether the student established that acetaminophen was administered. The second item reported whether the dosage of acetaminophen was established, while the third item recorded whether the student explicitly noted to the SP that the dosage was too high (Table 2).

**Table 2:** **Standardized patient checklist items related to medication error**

If student asks:	The SP responds:
Does anything seem to help him?	I have been giving him Tylenol but it doesn’t seem to help. (mother produces the bottle of Tylenol from her purse).
Are you giving any medications?	
What are you doing at home for him?	
How much are you giving him? Are you following the dosage instructions on the bottle?	We are giving 2 teaspoonfuls. My mom has been giving it to him several times a day since he has been sick (5 days) but it doesn’t seem to be helping him.
I think this dosage may be too much. You are giving him too much. (The learner acknowledges that you are giving Michael too much or the wrong dosage or simply addresses the dosage).	I didn’t realize I was giving him too much.

Additionally, students were asked to document their history and physical findings (gathered from the partial task trainer and video) and then to write an assessment and plan for the encounter. The assessment and plan was reviewed by 2 faculty members and included a score for whether the student identified the medication error anywhere in their documentation.

### Data analysis

All of the collected information was transferred to a database created for this case in Excel. Data analysis was performed using Stata, version 9.2 (StataCorp LP, College Station, TX). Frequencies and simple means were then calculated where appropriate. Chi-square analysis and *t* tests were used to test differences between groups.

## Results

During the study period 340 students completed this simulation. This represents all the medical students who were eligible during this time. A majority of students (69%;234/340) established that acetaminophen was being administered to the child while about half asked about the dose of acetaminophen (47%;161/340). Only 11% of all students completing the encounter (37/340) verbalized that the dose was too high to the SP during the encounter. Thirty four of these 37 students subsequently documented that the dosage was too high in their post-encounter structured note (Table 3). All of the students who identified that the dose was too high in their note also had made a verbal statement during the encounter. Thus 10% (34/340) of all students who completed this simulation documented the medication error. There was no significant difference between the performance of male and female medical students. Eighty-six percent of students (291/340) had completed the patient safety curriculum prior to the CCSE. Ten percent (30/291) of these students noted the overdose in their documentation while 14% (7/49) of the students that hadn’t yet completed the patient safety exercise noted the over dosage. These groups were not statistically different (p = 0.41). Students who identified the dosing error scored higher on the OCSE (mean score 76%) compared to those that did not (mean score 67%) and this was statistically significant (p < .01), though their overall performance on the CCSE was not different (73% compared to 72%; p = .20).

**Table 3:** **Student performance: identification of medication administration error**

Academic year	Number of students	Verbally noted by student during simulation* N(%)			Written in note after simulation N(%)		
		Student discovered that acetaminophen was administered n(%)	Student established the dosage of acetaminophen	Student stated that the dose was too high	Student identified medication error in write up	Male	Female
2010-11	114	88 (77)	44 (39)	12 (11)	12 (11)	4 (4)	8 (7)
2011-12	108	44 (41)	49 (45)	9 (8)	9 (8)	8 (7)	1 (1)
2012-13	118	102 (86)	68 (58)	16 (14)	13 (11)	7 (6)	6 (5)
Total	340	234 (69)	161 (47)	37 (11)	34 (10)	19 (6)	15 (4)

*recorded by standardized patients immediately upon completion of the encounter.

## Discussion

To our knowledge, this is the first study to evaluate the use of simulation as a modality to evaluate the abilities of medical students to identify dosing errors of over-the-counter medications. Only one prior study reported upon the use of an OSCE to assess other patient safety competencies such as risk factor identification and error reporting [13]. Additionally, another study analyzed the ability of medical students to identify prescribing errors using simulation-based methods [12]. In that study, only 11% of medical students correctly identified the embedded prescribing errors despite being prompted to search for them. In this setting we did not specifically prompt our students to search for medical errors yet our finding that 11% of medical students noted the overdose of acetaminophen is remarkably similar.

Patient harm from medication is common in the pediatric ambulatory setting with errors in parental medication administration resulting in the majority of preventable adverse drug events [15]. This is in contrast to pediatric inpatients where the majority of errors occur during the drug prescribing process [16]. Correct dosing in both settings is challenged by a myriad of factors including the need for weight-based dosing and conversion to volumes as many children require liquid preparations.

Because of the near ubiquity of acetaminophen as a pediatric medication, and the potential serious consequences of overdosage, we felt it would be an excellent marker for risk of medication error in our OSCE. We found that student performance was generally poor and did not differ between those who had previously completed a patient safety curriculum focused on medication administration errors and those who had not. It is possible that some students felt that such a dosing error is unlikely to be clinically significant, though we would note a case report of an 800 mg dose administered to an infant following a circumcision resulting in hepatotoxicity [17], and even underdosing holds the potential for clinical significance. It may also be that the skills that they learned in the preceding year extinguish quickly and thus argue for ongoing curricular efforts and continued reassessment of skills. Alternatively, they may have felt that the OSCE was an exercise focused primarily on clinical reasoning and making an accurate diagnosis and, because the symptoms of the child in the simulation did not overlap with those of acetaminophen toxicity, students may not have felt that the dosing was relevant reflecting their prioritization of the information they were collecting. We acknowledge that this is a complex OSCE case reflecting the complexities of actual clinical care and may be too advanced for such novice learners. Nevertheless, we were disappointed to see such a low number of students identify the medication error and equally disappointed that a third of students didn’t even determine that acetaminophen was being administered, particularly given our existing curriculum surrounding patient safety [11,18]. We suspect that medical students are much less likely to survey for OTC medications due to the perception that such medications are generally safe and efficacious, despite mounting evidence to the contrary, particularly in pediatrics [19]. Interestingly, half the students (161/340) established the dosage of acetaminophen being administered but only 23% (37/161) noted that this dose was too high either to the SP or in their documentation. This may suggest that we teach our students how to obtain a medication history without considering dose appropriateness. Our student participants were less than one year away from starting internship and our results suggest that additional training is needed if we hope to ensure better surveillance for medication errors occurring in the outpatient setting and particularly if they are over-the-counter medications.

While there are likely to be systems level prevention measures to prevent dosing errors, such as stronger packaging warnings, improved labeling and increased awareness, there remains a need for improving frontline healthcare providers’ skills in identifying and mitigating such errors [20]. Alternatively, it is perhaps not realistic to expect that frontline providers will be able to identify such errors while simultaneously tending to the complexities involved in the medical encounter and a robust patient safety system should include other checks to detect such errors. Nevertheless, our study findings support the need for additional medication error-identification and therapeutics education for medical students. Clerkships are the first opportunity to observe drug administration practices and to participate in the identification and prevention of medication administration errors. We suspect that improving students’ error-identification abilities would likely yield better prescribing practices, thereby producing better patient outcomes.

Our study has several limitations. Our findings represent the experience of a single medical school and these findings may not generalize to other programs. Although we compared two groups of students, we did not randomize them. However, both performed equally poorly. It is also possible that students did in fact note that the acetaminophen dosage was too high, but decided not to mention it during the encounter or during the write-up as they may have thought that a several-fold dosing error was insignificant.

## Conclusions

In this study, 89% of medical students failed to note a medication administration error of acetaminophen during a standardized patient simulation whether or not they completed a prior patient safety curriculum on medication administration errors. Students may not be adequately prepared to detect medication errors during internship. Further research is needed to determine the most appropriate teaching methods to increase medical students’ abilities to identify such medication administration errors.

## References

[1] Tzimenatos L, Bond GR, Pediatric Therapeutic Error Study Group (2009). Severe injury or death in young children from therapeutic errors: a summary of 238 cases from the American association of poison control centers. Clin Toxicol (Phila).

[2] Zandieh SO, Goldmann DA, Keohane CA, Yoon C, Bates DW, Kaushal R (2008). Risk factors in preventable adverse drug events in pediatric outpatients. J Pediatr.

[3] Frush KS, Luo X, Hutchinson P, Higgins JN (2004). Evaluation of a method to reduce over-the-counter medication dosing error. Arch Pediatr Adolesc Med.

[4] Simon HK, Weinkle DA (1997). Over-the-counter medications. Do parents give what they intend to give?. Arch Pediatr Adolesc Med.

[5] Bronstein AC, Spyker DA, Cantilena LR, Green JL, Rumack BH, Heard SE (2008). 2007 annual report of the american association of poison control centers’ national poison data system (NPDS): 25th annual report. Clin Toxicol (Phila).

[6] Samuels-Kalow ME, Stack AM, Porter SC (2013). Parental language and dosing errors after discharge from the pediatric emergency department. Pediatr Emerg Care.

[7] James L, Sullivan JE, Roberts D (2011). The proper use of acetaminophen. Paediatr Child Health.

[8] US Food and Drug Administration. Briefing information for the June 29–30, 2009, joint meeting of the drug safety and risk management advisory committee with the anesthetic and life support drugs advisory committee and the nonprescription drugs advisory committee. Available at: http://www.fda.gov/AdvisoryCommittees/CommitteesMeetingMaterials/Drugs/DrugSafetyandRiskManagementAdvisoryCommittee/ucm161515.htm. Accessed October 7, 2014.

[9] FDA drug safety communication. Addition of another concentration of liquid acetaminophen marketed for infants. Available at: http://www.fda.gov/Drugs/DrugSafety/ucm284741.htm. Accessed October 7, 2014.

[10] Miller MR, Robinson KA, Lubomski LH, Rinke ML, Pronovost PJ (2007). Medication errors in paediatric care: a systematic review of epidemiology and an evaluation of evidence supporting reduction strategy recommendations. Qual Saf Health Care.

[11] Dudas RA, Bundy DG, Miller MR, Barone M (2011). Can teaching medical students to investigate medication errors change their attitudes towards patient safety?. BMJ Qual Saf.

[12] Warholak TL, Queiruga C, Roush R, Phan H (2011). Medication error identification rates by pharmacy, medical, and nursing students. Am J Pharm Educ.

[13] Daud-Gallotti RM, Morinaga CV, Arlindo-Rodrigues M, Velasco IT, Martins MA, Tiberio IC (2011). A new method for the assessment of patient safety competencies during a medical school clerkship using an objective structured clinical examination. Clinics (Sao Paulo).

[14] Boulet JR, McKinley DW, Whelan GP, Hambleton RK (2003). Quality assurance methods for performance-based assessments. Adv Health Sci Educ.

[15] Kaushal R, Goldmann DA, Keohane CA, Christino M, Honour M, Hale AS (2007). Adverse drug events in pediatric outpatients. Ambul Pediatr.

[16] Kaushal R, Bates DW, Landrigan C, McKenna KJ, Clapp MD, Federico F (2001). Medication errors and adverse drug events in pediatric inpatients. JAMA.

[17] Ogilvie JD, Rieder MJ, Lim R (2012). Acetaminophen overdose in children. CMAJ.

[18] Thompson DA, Cowan J, Holzmueller C, Wu AW, Bass E, Pronovost P (2008). Planning and implementing a systems-based patient safety curriculum in medical education. Am J Med Qual.

[19] Sharfstein JM, North M, Serwint JR (2007). Over the counter but no longer under the radar–pediatric cough and cold medications. N Engl J Med.

[20] Reason J (2004). Beyond the organisational accident: the need for “error wisdom” on the frontline. Qual Saf Health Care.

